# Supplementary material for: Plasma-activated water promotes and finely tunes arbuscular mycorrhizal symbiosis in Lotus japonicus
Source: BMC Plant Biol. 2025 Apr 25;25:544. doi: 10.1186/s12870-025-06563-1 (PMC12032643; doi:10.1186/s12870-025-06563-1)
Supplement: Supplementary file 1 — Supplementary Material 1: Fig. S1. Phenotypic evaluation of mycorrhizal colonization after 4 weeks (early phase) in L. japonicus plants inoculated with the AM fungus R. irregularis after repeated irrigations with PAW 10’ or H2O only. Fig. S2. Viability of L. japonicus cell suspension cultures treated for either 1 h (A) or 48 h (B) with PAW 10’. Fig. S3. Inorganic phosphate (Pi) content in leaves of non-mycorrhizal L. japonicus seedlings after repeated irrigations with PAW 5’, PAW 10’ or H2O only. Fig. S4. Quantification of nitrogen, carbon and sulphur content in dry shoots of non-mycorrhizal L. japonicus seedlings after repeated irrigations with PAW 5’, PAW 10’ or H2O only. Fig. S5. Representative image of the generation of plasma-activated water (PAW) by exposing water to a plasma torch. [file 12870_2025_6563_MOESM1_ESM.docx]

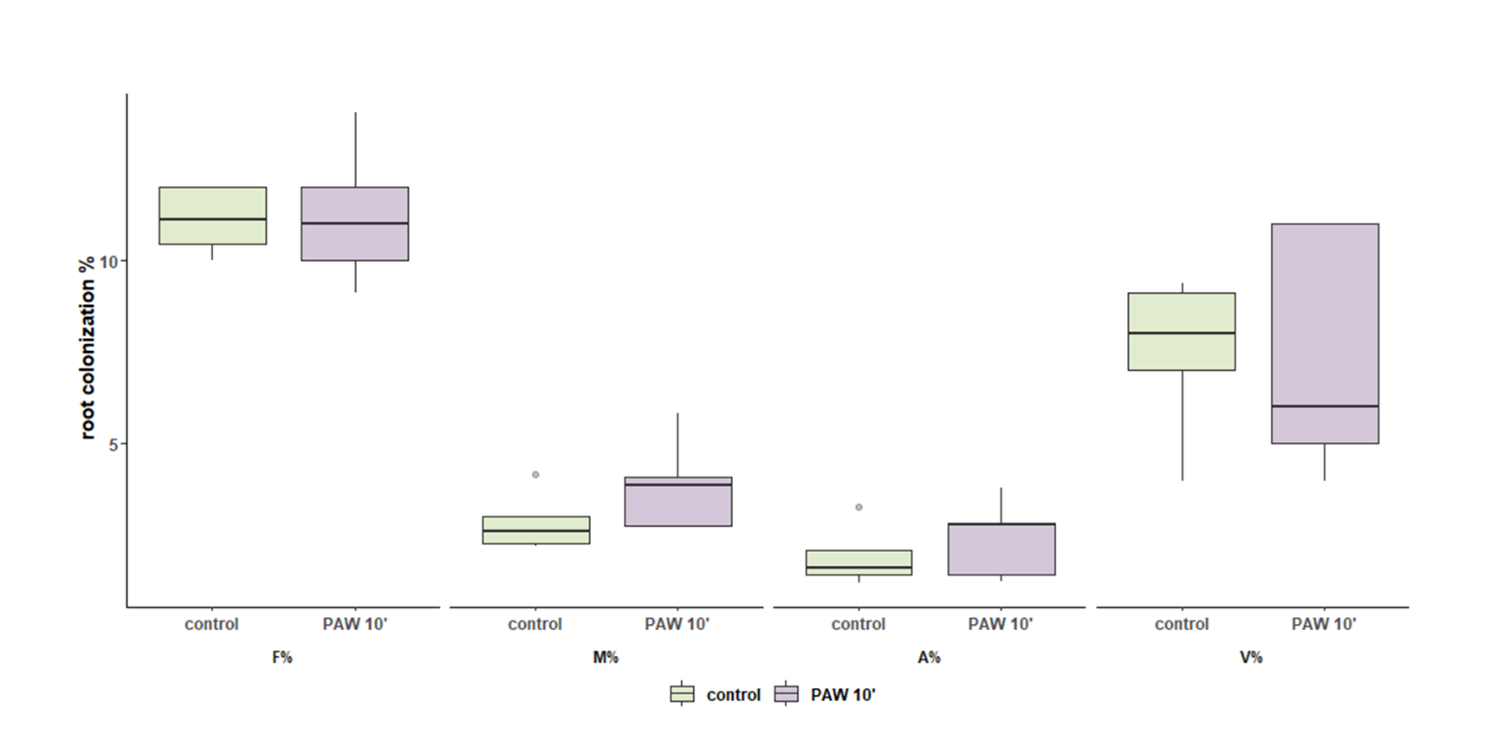


**Fig. S1.** Phenotypic evaluation of mycorrhizal colonization after 4 weeks (early phase) in *L. japonicus* plants inoculated with the AM fungus *R. irregularis* and irrigated twice per week alternating low phosphate modified Long-Ashton with PAW 10’ (light purple) or H_2_O (control, light green). PAW was obtained by exposing deionized H_2_O for 10 min to atmospheric plasma generated by a plasma torch operating at 900 W. The quantification of the degree of mycorrhization was conducted via the Trouvelot method. F%, frequency of mycorrhiza; M%, intensity of the mycorrhizal colonization; A%, arbuscule abundance. Vesicle abundance (V%) was also determined. Data are shown as boxplots of 5 biological replicates. No statistical differences could be identified by ANOVA.


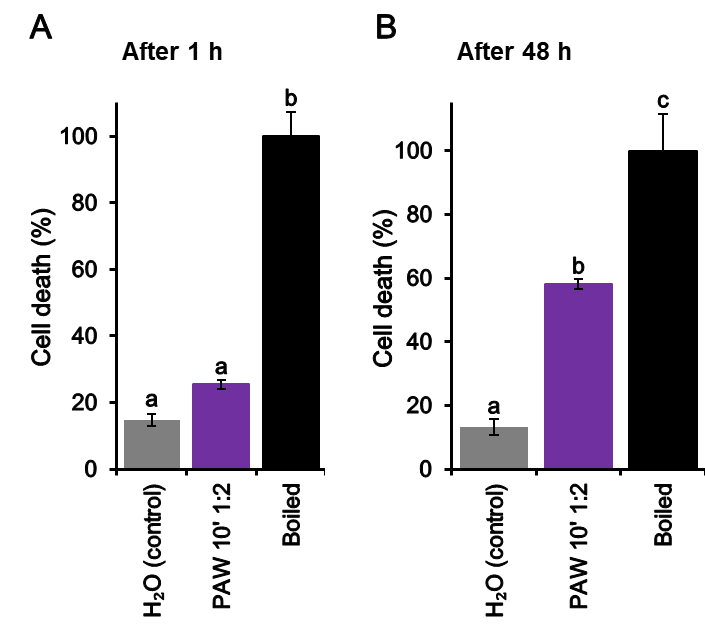


**Fig. S2.** Viability of *L. japonicus* cell suspension cultures treated for either 1 h (A) or 48 h (B) with PAW 10’. PAW was generated by exposing deionized H_2_O to plasma torch-derived atmospheric plasma for 10 min at 900 W 1:2 diluted (purple). Control cells were incubated with cell culture medium only (grey). The 100% value corresponds to cells treated for 10 min at 100°C (black bars). Data are the means ± SE of 3 biological replicates. Bars labelled with different letters differ significantly (*p* < 0.05, ANOVA test followed by Tukey’s HSD).


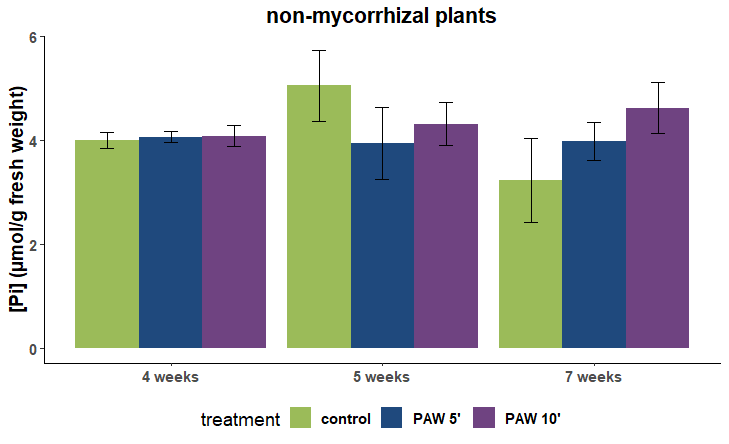


**Fig. S3.** Inorganic phosphate (Pi) content in leaves of non-mycorrhizal *L. japonicus* seedlings repeatedly irrigated with PAW 5’, PAW 10’ or H_2_O. Pots containing *L. japonicus* seedlings were irrigated twice per week alternating low phosphate modified Long-Ashton with either PAW 5’ (blue), PAW 10’ (violet) or H_2_O(control, green) and harvested after 4 (early phase), 5 (mid phase) or 7 weeks (late phase) after the first treatment, respectively. PAW was obtained by exposing deionized H_2_O for either 5 min or 10 min to atmospheric plasma generated by a plasma torch operating at 900 W. Data are the means ± SE of 5 biological replicates. No statistical differences among groups could be identified by ANOVA.


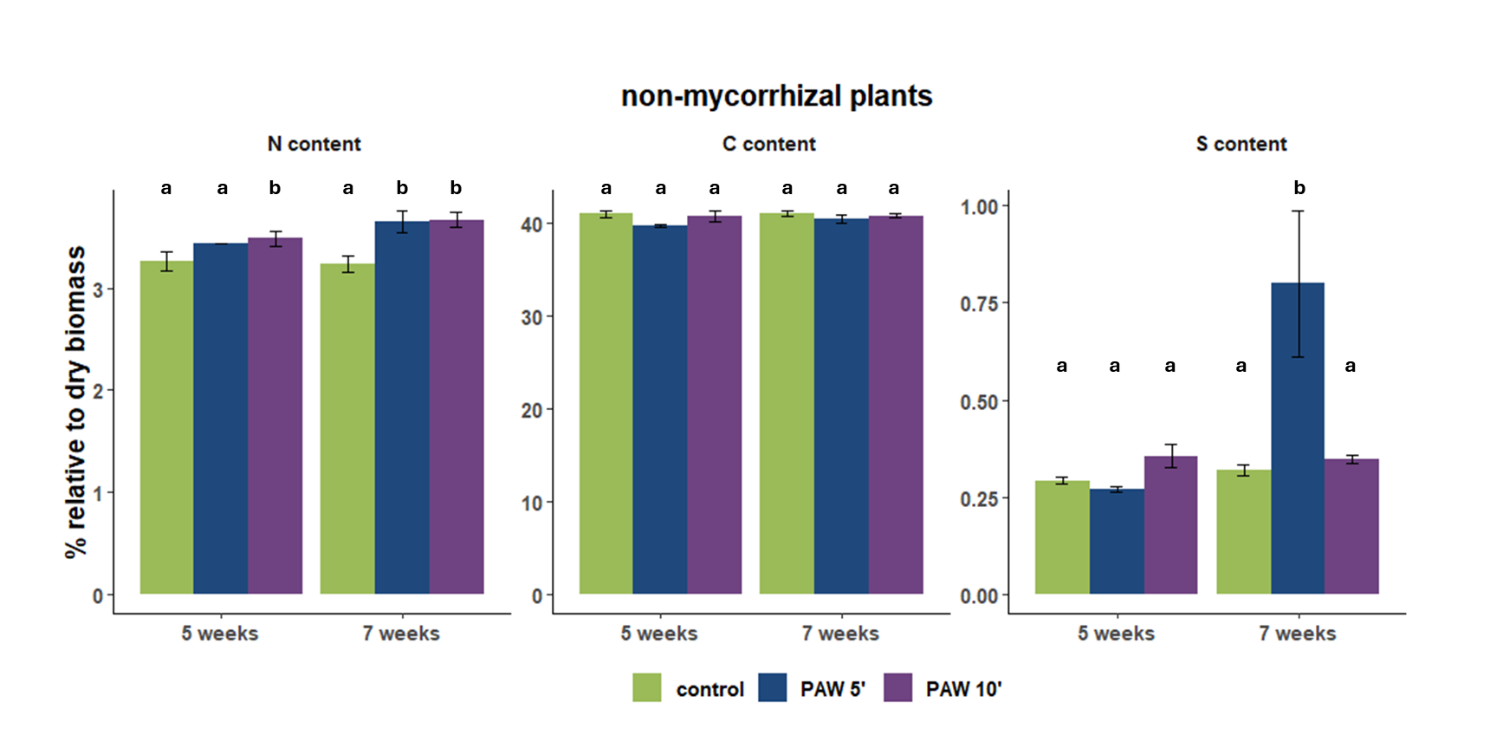


**Fig. S4.** Quantification of nitrogen, carbon and sulphur content in dry shoots of non-mycorrhizal *L. japonicus* seedlings. Pots containing *L. japonicus* seedlings were irrigated twice per week alternating low phosphate modified Long-Ashton with either PAW 5’ (blue), PAW 10’ (violet) or H_2_O (control, green) and harvested after 5 (mid phase) or 7 weeks (late phase) after the first treatment, respectively. PAW was obtained by exposing deionized H_2_O for either 5 min (PAW 5’) or 10 min (PAW 10’) to atmospheric plasma generated by a plasma torch operating at 900 W. The content of nitrogen, carbon, sulphur is expressed as percentage relative to the dry shoot biomass (% w/w). Data are the means ± SE of 5 biological. Bars labelled with different letters differ significantly (*p* < 0.05, ANOVA followed by Tukey’s post-hoc).


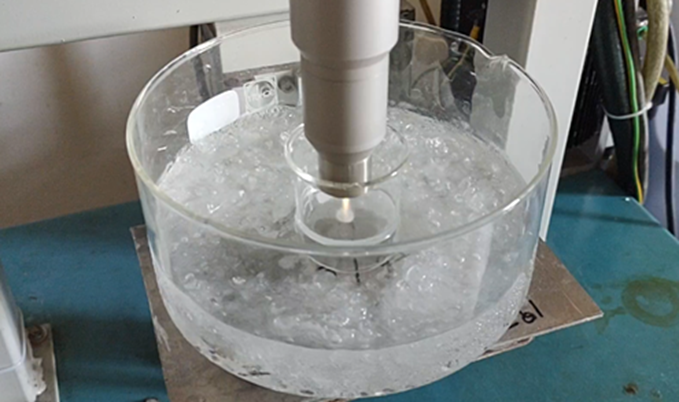


**Fig. S5.** Representative image of the generation of plasma-activated water (PAW) by exposing water to a plasma torch.
